# Exploring Primary Healthcare Needs Among Migrants in Australia: A Scoping Review

**DOI:** 10.3390/healthcare14193168

**Published:** 2026-09-24

**Authors:** Leah East, Rebecca M. Jedwab, Jessica Elliott, Vainess Mbuzi, Liz Ryan, Aletha Ward, Amornrat Saito, Anthea Fagan, Tao Wang

**Affiliations:** 1 School of Nursing and Midwifery, University of Southern Queensland, Toowoomba, QLD 4350, Australia; rebecca.jedwab@unisq.edu.au (R.M.J.); liz.ryan@unisq.edu.au (L.R.); aletha.ward@unisq.edu.au (A.W.); alison.wang@ecu.edu.au (T.W.); 2 Institute for Health, University of Southern Queensland, Springfield, QLD 4300, Australia; jessica.elliott@unisq.edu.au (J.E.); vainess.mbuzi@unisq.edu.au (V.M.); 3 School of Health, University of New England, Armidale, NSW 2350, Australia; 4 School of Nursing and Midwifery, University of Southern Queensland, Ipswich, QLD 4305, Australia; amornrat.saito@unisq.edu.au; 5 School of Nursing and Midwifery, Griffith University, Nathan, QLD 4111, Australia; 6 Faculty of Health, Southern Cross University, Coffs Harbour, NSW 2450, Australia; anthea.fagan@scu.edu.au; 7 School of Nursing and Midwifery, Edith Cowan University, Joondalup, WA 6027, Australia

**Keywords:** Australia, culturally responsive care, health equity, health literacy, healthcare access, migrants, primary healthcare

## Abstract

**Highlights:**

**What are the main findings?**

- Migrants require targeted healthcare strategies that promote health literacy and navigation towards primary healthcare services that meet specific needs.
- Health promotion campaigns that reduce stigma and promote avenues for healthcare associated with sensitive issues including sexual and reproductive health are required.

**What are the implications of the main findings?**

- Socialisation of healthcare professionals and services that extend beyond general practice could enhance healthcare access and treatment, providing a more comprehensive approach to meeting the healthcare needs of migrants.

**Abstract:**

**Background/Objectives:** Australia is a multicultural country; however, migrants encounter multiple barriers in accessing healthcare services that can detrimentally impact timely healthcare, leading to poor healthcare outcomes. Recognising that primary healthcare services are often the first point of contact for migrants in Australia, the aim of this scoping review was to explore and synthesise the existing evidence on migrants’ primary healthcare needs and associated experiences of accessing and engaging with primary health services within the Australian healthcare context. **Study type:** A scoping review was conducted in accordance with the Joanna Briggs Institute methodology and reported following the Preferred Reporting Items for Systematic Reviews and Meta-Analyses extension for Scoping Reviews (PRISMA-ScR). **Methods:** Seven databases were searched to identify peer-reviewed primary research studies published in English between 2013 and 2025 that examined adult migrants’ primary healthcare needs and associated experiences in Australia. Data were synthesised using thematic analysis and narrative synthesis. **Results:** Twenty-eight studies were included. Migrants’ primary healthcare needs centred on improving health literacy, navigating the Australian healthcare system, culturally responsive communication, and access to preventative care and condition-specific health services. Key barriers included language and communication difficulties, limited health literacy, and cultural beliefs and stigma. Trusted relationships with healthcare providers, culturally responsive models of care and community support were identified as enablers of access to and engagement with primary healthcare. **Conclusions:** Migrants’ primary healthcare needs in Australia extend beyond access to clinical services and include health literacy, culturally responsive care, and support to navigate the healthcare system and appropriate preventative health services. The findings highlight opportunities to strengthen primary healthcare policy and practice through improved communication, interpreter access, health literacy initiatives, continuity of care, and community-informed health promotion strategies that are responsive to the diversity of Australia’s migrant communities.

## 1. Background

Migration has played a significant role in shaping Australia’s cultural, ethnic, and linguistic diversity. Australia is a culturally diverse nation, with 8.8 million overseas-born residents accounting for 32% of the population as of 30 June 2025 [1]. In this review, the term migrant refers to a person who has moved from their country of origin to establish residence in Australia. Overseas migration continues to be the primary contributor to Australia’s population growth [1], with thousands of new arrivals each year. In the Australian context, the terms “migrants” and “immigrants” are often used interchangeably; in this review, both refer to individuals who moved from their country of origin into Australia to establish residence [2]; however, for the purposes of this review, we have used the term migrants for consistency.

Migrants from culturally, ethnically, and linguistically diverse backgrounds can experience significant barriers in accessing healthcare, leading to underutilisation of primary and preventive care services [3] and poorer health outcomes compared to the Australia-born population [4]. These barriers include communication difficulties, conflicting sociocultural values and beliefs, limited health literacy, and a lack of culturally responsive healthcare services [5]. Inadequate access to timely healthcare may contribute to undiagnosed or poorly managed chronic conditions and greater reliance on emergency services for conditions that could be managed through timely primary healthcare [3,5].

Primary healthcare is a foundation of an effective healthcare system and is often the first point of contact within Australian healthcare [6]. Primary healthcare provides essential healthcare services such as general practice medicine, community health, and preventive care, which are vital for early disease detection, management of chronic conditions, and health promotion [7]. Accessible and culturally responsive primary healthcare is particularly important for migrants across Australia’s diverse geographical landscape, where hospital and specialist services may be limited [8]. Enhancing primary healthcare access and utilisation across diverse geographical landscapes is recognised as a cost-effective strategy to improve migrant health outcomes and reduce emergency service dependence [3,9]. Understanding migrants’ healthcare experiences and preferences can inform the development of culturally sensitive care, ensuring both competence and positive health outcomes [10].

A clearer understanding of migrants’ primary healthcare needs can inform future research and support the development of accessible and culturally responsive primary healthcare services, workforce capability, and models of care. This evidence is needed to strengthen policy and practice focused on improving access, quality and equity in primary healthcare for Australia’s diverse migrant communities.

A growing body of research has examined migrants’ experiences of accessing and utilising healthcare services in Australia [11,12,13,14,15,16]. These studies identify challenges that migrants face when seeking healthcare, including long waiting times for public health services, communication difficulties, limited familiarity with the Australian healthcare system, and services that do not adequately respond to migrants’ needs. Several systematic and scoping reviews have synthesised evidence regarding migrants’ healthcare access and utilisation. Two reviews [6,17] focused on primary healthcare. Patel et al. reviewed communication challenges faced by refugees and asylum seekers in primary healthcare consultations based on 21 included studies [6]. Kamran et al., through a scoping review of 46 studies, identified multiple barriers that prevented migrants from accessing primary healthcare, such as ethnic discrimination, language difficulties, and socioeconomic constraints [17]. However, neither review focused specifically on the Australian context. Furthermore, no subgroup analyses were conducted across different health systems. As a result, the applicability of these findings to migrants living in Australia is uncertain.

Two scoping reviews [5,18] examined migrants’ experiences within the Australian healthcare system; however, neither specifically focused on primary healthcare. Therefore, this review specifically synthesises evidence relating to migrants’ primary healthcare needs, associated experiences, and engagement with primary healthcare services in Australia and identifies primary healthcare-specific priorities. The specific objectives were: (a) to explore migrants’ primary healthcare needs and associated experiences of accessing and engaging with primary healthcare services in Australia, and (b) to provide suggestions for future research, policy and practice to address identified migrant-specific service gaps.

## 2. Methods

### 2.1. Design

This scoping review was conducted to map and synthesise the primary healthcare needs and associated experiences of migrants across Australia. The review followed the scoping review methodological frameworks comprising five stages: (1) identifying the research question; (2) identifying relevant studies; (3) selecting studies; (4) charting and analysing the data; and (5) reporting the findings [19,20]. The Preferred Reporting Items for Systematic reviews and Meta-analyses extension for Scoping Reviews (PRISMA-ScR) checklist was followed to enhance reporting quality and transparency [21] (Table S1).

Stage 1: Identifying the research question

The primary question developed by the research team was: What are the primary healthcare needs of migrants in Australia?

Stage 2: Identification of relevant studies

Eligibility criteria were guided by the Population–Concept–Context (PCC) framework [22]. This framework was selected as the review aimed to explore and map the breadth of the literature rather than evaluate the effectiveness of specific interventions [23]. For this review, primary healthcare needs were not pre-defined but were identified inductively from the included studies. Healthcare needs were considered to include the information, services, supports, and models of care required to access and engage with primary healthcare. Associated healthcare experiences, barriers, enablers, and preferences were explored as influencing factors determining whether primary healthcare needs were met. Studies were included if they: Examined migrants’ needs, access to or utilisation of components of any primary healthcare within Australia inclusive of associated experiences; were peer-reviewed primary research (qualitative, quantitative or mixed-methods designs); and published in English from 2013 (to align with the National Primary Health Care Strategic Framework [24]) to 2025. The full inclusion and exclusion criteria related to the PCC framework components are presented in Table 1.

### 2.2. Search Strategy

Seven electronic databases (CINAHL, MEDLINE, PSYCINFO, SCOPUS, JBI, COCHRANE, INFORMIT) were searched on 1 August 2025. The search strategy was developed and tested in consultation with an experienced health services librarian from the University of Southern Queensland.

Medical Subject Headings (MeSH) and keywords were used in combination with Boolean operators (AND, OR) to refine the search. A three-phase search strategy was applied: (1) An initial search of two databases to identify relevant keywords and index terms; (2) A comprehensive search across all databases using identified terms; and (3) Screening of reference lists of included studies to identify additional articles. All results were documented and imported into the software referencing management system EndNote. The search strategy for MEDLINE was: (MH “Emigrants and Immigrants” OR XB Migrant* OR XB Immigrant*) AND (MH “Primary Health Care” OR XB “primary healthcare” OR XB “primary health care” OR XB “primary care” OR XB GP OR XB “general practitioner*” OR XB “community health*”) AND Australia*, with the limiters English language and years 2013 to 2025.

Stage 3: Study selection

All retrieved articles were imported into EndNote and duplicates removed. Records were then uploaded to Covidence for screening. Two research team members independently screened each article at the title and abstract levels (L.E., R.J., J.E., V.M., L.R., T.W.), followed by full-text review against the inclusion criteria (R.J., J.E., V.M., L.R.). Reasons for exclusion were documented at the full-text stage. Disagreements were resolved through discussion, with a third reviewer adjudicating where required. The study selection process is presented in Figure 1 using a Preferred Reporting Items for Systematic Reviews and Meta-analyses (PRISMA) flow diagram [21].

Stage 4: Data extraction and analysing the data

Data extraction was undertaken independently by two members of the research team within Covidence (R.J., J.E.). A data extraction tool was developed by the research team to systematically collect relevant information from the included studies. Extracted data included details on the population, concept, context, methodology, and key findings. Disagreements among reviewers were resolved through discussion.

Thematic analysis was supported by narrative synthesis to map the key findings [25]. Coding was undertaken independently by multiple reviewers (L.E., R.J., J.E., A.F.). Themes were discussed and refined through discussion with the broader research team to enhance analytical rigour. Themes related to migrants’ primary healthcare needs, healthcare experiences, barriers, enablers, and preferences were identified through an iterative process of reading, coding, and categorisation. Thematic analysis was selected for its flexible yet structured approach, allowing integration across diverse study designs and data types [26].

Stage 5: Reporting the findings

Findings were collated and presented as a narrative synthesis aligned with the review question. Four main themes were developed: (1) The Crucial Role of the General Practitioner; (2) Language, Communication and Health Literacy; (3) Preventative Healthcare Needs; and (4) Cultural Beliefs, Stigma, Fear, and the Positive Influence of Community. Furthermore, findings were organised by study characteristics, key findings, and identified gaps in the literature. The review provides a comprehensive overview of migrants’ primary healthcare needs and associated healthcare experiences, including the barriers, enablers, and preferences that influenced access to and engagement with primary healthcare services.

## 3. Results

A total of 28 studies were included, the majority of which were qualitative studies [27,28,29,30,31,32,33,34,35,36,37,38,39,40,41,42,43], while nine were quantitative [44,45,46,47,48,49,50,51,52] and two used mixed methods [53,54]. Most studies were conducted in metropolitan areas and major capital cities of Australia including Melbourne [28,37,40,49], Sydney [32,39,43], Brisbane [41,50,52], and Adelaide [34]. However, some studies specified the state instead of the city, for example, Queensland [36,44,45], New South Wales [27,35,38], Victoria [29,30,31], and eight studies were Australia-wide [33,42,46,47,48,51,53,54]. The country of origin of migrants varied across the studies. The majority were mixed [28,29,30,31,32,33,38,39,44,45,46,47,49,51,52,54], with other countries or areas specified including: India [27,53], Vietnam [40], China [42,43,48], Greece [34], Japan [35], Africa [37], Thailand [41], and Latin America [50]. Refer to Table S2 for study characteristics.

### 3.1. The Crucial Role of the General Practitioner

All studies included use of General Practitioner (GP) services, most commonly in conjunction with other community-based services including community centres, nursing, and/or specialist services as required. Services varied from routine and preventative care [34,38,46,48] to condition-specific or focused needs, including: mental health and chronic diseases [32,41,42,47], asthma [28], sexual and reproductive health [33,35,36,37,44,45,53], liver (hepatitis) [39,52], cardiometabolic and cardiovascular [43,49,50], oral health [51], women’s health [40,54], and diabetes [27,29,30,31]. The GP therefore functioned as the primary gateway to the broader healthcare system. Establishing a relationship with a regular, trusted GP was a critical enabler of care. For example, Indian migrant men reported a strong preference for consulting their regular GP about sensitive issues, including sexual health, highlighting the importance of continuity and trust [53]. Similarly, a study of Latin American migrants demonstrated that culturally responsive primary healthcare models, such as specialised clinics offering extended consultations, effectively supported management of complex chronic conditions and comorbidities [50].

Despite this, many migrants needed more information on the role and scope of a GP and what other services were available beyond this [38,50,51,53]. Many were unaware of what screening GPs could do or what referrals to other specialists could be obtained. Participants in some studies indicated that the education and information that was provided needed to be culturally appropriate and understandable. For many participants, this meant providing information in their language of origin or from GPs with the same ethnic background [41,43,51]. However, this was dependent on the healthcare needs of migrants. For example, Inoue et al. found that migrants did not want to speak to health service providers from the same cultural background around contraception and sexual health issues because of the potential shame and stigma associated with these sensitive issues and healthcare needs [35]. Similarly, participants in Ramanathan et al.’s study expressed the desire for the availability of information associated with sexual health to be available within an anonymous context [53], and Istiko et al. found that in addition to seeking health information from trusted GPs, peers, partners and those who have lived experience were utilised for HIV health literacy [36].

Culturally safe healthcare engagement and rapport with healthcare professionals was also a recognised need among migrants [32,36,43,54], which had the ability to influence perceived health conditions [47]. For example, Hu and Wang found that Chinese migrants who had higher self-reported satisfaction levels with a GP and awareness of professional healthcare for mental health issues had a lower risk of psychological distress [47]. Similarly, Chinese migrants were less likely to self-medicate with antibiotics if they were satisfied with healthcare services and had access and no perceived barriers to primary healthcare services [48]. Moreover, migrants’ primary healthcare needs and experiences were closely intertwined with the barriers and enablers that influenced whether those needs were being met.

### 3.2. Language, Communication and Health Literacy

Access to care was significantly enhanced when migrants could engage with language-concordant providers and draw on family and community support networks. The ability to communicate in one’s preferred language reduced communication barriers and fostered trust and understanding. For instance, Chinese migrants with cardiovascular disease expressed a preference for Mandarin-speaking GPs and specialists [43], while Vietnamese women sought female, Vietnamese-speaking GPs for culturally sensitive discussions [40]. Language and communication barriers were also the most pervasive issues identified across migrant groups. Migrant populations were missing information around the specific health condition or treatment [27,31,44], but in addition to this, needed information associated with navigating health systems and services that were available to them [28,34,37,38].

Limited English proficiency extended beyond basic communication difficulties to challenges in navigating complex administrative systems, articulating symptoms, and understanding nuanced health information. For example, a study on older Vietnamese-born women found that although participants could follow simple procedural instructions during mammography, their inability to ask questions or understand complex follow-up information left them feeling disempowered and inadequately informed [40]. These challenges were further exacerbated by the rapid shift to telehealth during the COVID-19 pandemic. Older Chinese migrants, who might have relied on gestures or family support during in-person consultations, experienced significant difficulties communicating effectively with GPs via telephone consultations, resulting in missed appointments and unresolved health issues [42].

In addition to language barriers, many migrants faced challenges related to health literacy. These challenges manifested in two distinct ways: limited awareness of health conditions and prevention screening, and difficulty navigating the Australian healthcare system. For example, a study examining culturally and linguistically diverse (CALD) populations with viral hepatitis identified substantial knowledge gaps and misconceptions associated with infection transmission [52]. Notably, only 6% of participants understood the link between hepatitis and liver cancer and the importance of ongoing surveillance [52]. Similarly, research involving Indian migrants highlighted limited awareness of oral cancer risks factors relevant to their community, such as areca (betel) nut use and alcohol consumption, along with poor recognition of early symptoms [51]. These gaps were not confined to older migrants. Younger adults from migrant backgrounds also reported that secondary education did not adequately equip them with practical knowledge of the health system, including how to access Medicare (Australian universal public health insurance scheme) or specialist services independently [38].

### 3.3. Preventative Healthcare Needs

Across multiple studies, preventative healthcare emerged as an important unmet need among migrant communities. Barriers included limited awareness of disease risk factors, screening programs, and preventative health services, as well as challenges navigating screening pathways. Low participation in preventative healthcare was reported in relation to cervical cancer screening [44,45], HIV testing and HIV health literacy [33,36], hepatitis knowledge and surveillance [39,52], oral cancer prevention [51], and women’s health services [40,54]. Migrants also reported limited knowledge of available screening programs and preventative healthcare pathways within the Australian healthcare system [38].

### 3.4. Cultural Beliefs, Stigma, Fear, and the Positive Influence of Community

Help-seeking behaviours were also influenced by cultural beliefs, stigma, fear, and the negative influence of community. In several communities, these factors acted as significant deterrents to accessing care. For example, among Arabic and Assyrian populations, fear of receiving ‘bad news’ and stigma associated with certain diagnoses discouraged health service engagement [39]. Cancer was sometimes referred to euphemistically as ‘the ugly disease’, and diagnoses were often concealed due to perceptions of shame or beliefs relating to retribution [39]. Likewise, the stigma associated with HIV delayed testing among African and Asian migrants [33]. Additionally, the integration of traditional health beliefs, such as Traditional Chinese Medicine, could complicate chronic disease management (for example, cardiovascular disease) by creating tensions with Western treatment approaches and delaying responses to acute symptoms [43]. In other cases, cultural perceptions of health processes as ‘natural’ reduced engagement with preventative care. For example, some Vietnamese women viewed menopause as a normal life stage not requiring medical consultation, thereby missing opportunities for health-promoting strategies [40].

Beyond the clinical setting, community played a vital role in supporting health and wellbeing. In one study, a Thai temple functioned as both a social hub and a source of emotional and practical support [41]. Similarly, Chinese community organisations facilitated group exercise programs and health education sessions, supporting chronic disease self-management [43]. Trusted, non-clinical communication channels, such as ethnic media, community radio, and word-of-mouth, were also effective in disseminating health information in culturally appropriate ways [38,39].

## 4. Discussion

This scoping review examined migrants’ primary healthcare needs and associated healthcare experiences within Australia across 28 studies. Across the included studies, healthcare needs were closely intertwined with healthcare experiences, and barriers, enablers, and preferences influenced whether migrants were able to access, engage with, and benefit from primary healthcare services. The findings suggest that migrants’ primary healthcare needs and associated experiences are shaped by four interconnected factors: (1) health literacy and system navigation, (2) culturally responsive communication, (3) trusting relationships and continuity of care, and (4) access to preventative healthcare services. Migrants required support to understand health information, navigate referral pathways and access preventative healthcare services. The findings suggest that health literacy extends beyond knowledge of health conditions and includes the practical ability to navigate Australia’s complex primary healthcare system, understand referral pathways and access preventative services. Key barriers included language and communication difficulties; low health literacy; limited awareness of health conditions and prevention screening; challenges navigating the Australian healthcare system; and cultural beliefs, stigma and fear that influenced help seeking. Conversely, trusted relationships with GPs, culturally responsive models of care, language-concordant communication and support from community organisations facilitated access to and engagement with primary healthcare.

Many of the barriers experienced by migrants arose through the interaction between individuals and the organisation of primary healthcare services. Language barriers, difficulties navigating the healthcare system, inconsistent access to interpreters and variation in culturally responsive care therefore need to be understood within a service context. Moezzi et al.’s review [9] of migrants and refugees in low- and middle-income countries similarly identified language and referral difficulties but emphasised financial constraints and shortages of trained healthcare staff. These differences in emphasis may reflect healthcare resources and financing arrangements, as well as the inclusion of refugees in that review [9]. The findings highlight the importance of adapting primary healthcare services to the needs of diverse migrant communities rather than adopting a one-size-fits-all approach. Although migrants are frequently considered as a single population group, the findings of this review demonstrate considerable variation in healthcare needs, communication preferences and barriers across different cultural communities. This variation suggests that ethnic or cultural background alone cannot explain an individual’s healthcare preferences or experiences. Policies and service models should therefore be sufficiently flexible to respond to local population needs.

Holistic primary healthcare recognises migrants’ individual health needs, cultural contexts, and preferences [55]. The communication and language barriers identified in this review are also supported by wider Australian research involving migrants with refugee backgrounds [56]. Internationally, Tsai and Ghahari’s Canadian scoping review similarly identified communication and cultural barriers to healthcare access [10]. The recurrence of these findings across settings suggests shared difficulties in providing accessible care across languages and cultural contexts. Thus, language-concordant care and culturally responsive communication strategies could include the use of interpreters and bilingual staff, digital translation tools and multilingual eHealth applications [57,58,59,60]. Health education materials such as brochures or pamphlets should also be easy to understand and available in multiple languages and formats [5,58].

Interpreter services need to be more easily available to assist in primary healthcare for migrants, both in person and on the phone [57]. Having a GP from the same ethnic or cultural background was found to facilitate better communication and a greater level of understanding between migrants and their GP and facilitated better primary healthcare access [60]. However, preferences for culturally or language-concordant providers were not universal, particularly when care involved sensitive or stigmatised health concerns. Having healthcare providers from similar backgrounds to service users can also help identify cultural variations and recognise cultural differences, which is important for effective service provision that can address stigma and discrimination [56]. However, some participants in the included studies preferred greater anonymity [35,53]. Related concerns were reported by migrant women in Geneva, some of whom were uncomfortable discussing sensitive issues with interpreters from their own communities [58]. Although that study focused specifically on reproductive healthcare; it helps explain why shared language or background may be valued alongside, rather than perceived privacy and emotional safety.

Non-judgemental and compassionate approaches are important to ensure that migrants are not deterred from accessing primary healthcare services [5]. Previous evidence on interpreter-mediated care also highlights the importance of trust and rapport in encouraging patients to share their needs and concerns with healthcare providers [61]. Singla et al. found that Indian migrants living with chronic illnesses valued compassionate and emotionally supportive care from their GPs, which facilitated trusting patient–provider relationships [60].

Culturally responsive communication can also help patients feel welcomed, heard, and respected, encouraging them to seek advice from healthcare providers, leading to better health outcomes [62]. These findings reinforce the importance of developing a primary healthcare workforce that is equipped to provide culturally responsive and person-centred care. This extends beyond understanding cultural differences to recognising the diverse experiences and healthcare needs within migrant communities and responding to these in partnership with patients [5,60,62].

Healthcare providers have a significant role in ensuring migrants have adequate access to primary healthcare services [9]. Limited access to understandable health information and unfamiliarity with the Australian healthcare system may reduce engagement with healthcare, health promotion and disease detection activities [5,63]. Improving health literacy should therefore encompass both knowledge of specific health conditions and the practical skills required to navigate healthcare services.

Beyond communication and health system navigation, the review identified preventative healthcare as a key unmet need among migrant communities. Multiple studies highlighted low participation in screening programs; limited awareness of risk factors and early disease symptoms; and challenges accessing preventive services related to cervical cancer [44,45], HIV [33,36], hepatitis [39,52], oral cancer [51] and women’s health [40,54]. Migrants also reported limited knowledge of available preventative services and screening pathways [38]. The findings suggest that preventative healthcare is often deprioritised when migrants face competing challenges associated with language barriers, limited understanding of the Australian healthcare system, uncertainty regarding available services, and cultural beliefs or stigma surrounding sensitive health conditions. Consequently, primary healthcare providers have an important role in promoting preventative healthcare through culturally responsive health promotion, opportunistic screening discussions and early intervention strategies. Improving awareness of available screening programs and preventative services may support earlier engagement with healthcare and help address barriers to preventative care identified across migrant communities [33,37,44,45,52].

The findings also highlight the importance of providing clear information about the role of GPs, referral pathways, preventative health services and available community supports. This may improve confidence in accessing care and encourage earlier engagement with primary healthcare services, particularly among migrants who have limited previous experience of the Australian health system [38]. There remains a clear need for targeted attention to preventative care and screening, including sexual and reproductive health, HIV and sexually transmitted infections, and cancer screenings. Stigma, cultural and religious beliefs, confidentiality concerns and limited awareness were identified as barriers that may influence whether migrants seek information, testing or care for sensitive health issues [33,37,39]. Routine consultations with GPs, nurse practitioners and other healthcare professionals provide opportunities to identify unmet screening needs, improve health literacy and facilitate timely referral to appropriate services, particularly for conditions where a delayed diagnosis or low screening participation has been identified among migrant communities [37,52].

An important finding of this review was the geographical concentration of the available evidence, with many studies conducted in metropolitan settings and limited evidence concerning migrants living in regional, rural and remote Australia. A similar urban emphasis in earlier migrant health research was noted by Bin Abdul Baten et al. in the United States [64]. Their rural–urban analysis identified greater cost-related difficulties among rural immigrants, despite their being more likely to report a usual source of care [64]. Zeng et al.’s rural review also highlighted transport difficulties and limited service availability [65]. These findings could explain why metropolitan evidence may not capture the full range of access barriers. Differences in healthcare financing and migrant populations limit direct transferability, and national or state-wide studies in our review do not necessarily provide separate evidence on rural experiences.

### Healthcare Policy, Practice and Research Implications

The findings of this review have important implications for primary healthcare policy, practice and research. Many of the barriers identified across the included studies are potentially modifiable through changes in service delivery, communication strategies, and models of care. Improving equitable access to primary healthcare will therefore require coordinated action across policy, health services and primary healthcare practice.

Priority areas include improving access to accredited interpreter services, providing multilingual health information, strengthening culturally responsive communication, and supporting continuity of care through ongoing relationships with trusted healthcare professionals. Services should also recognise that migrant communities are not homogeneous. Health promotion initiatives, preventative screening programs and models of care should be tailored to the health priorities, languages and cultural contexts of local communities wherever possible [5].

The review also highlights the importance of working collaboratively with migrant communities to ensure that health information, service delivery and health promotion strategies are acceptable, accessible, and responsive to community needs. Such approaches have the potential to strengthen health literacy, improve engagement with preventative healthcare and contribute to more equitable health outcomes. Future research should move beyond describing barriers towards evaluating interventions that improve access, health literacy and the effectiveness of culturally responsive primary healthcare models across diverse migrant communities. Research is also needed to explore how migrants access and utilise primary healthcare services beyond metropolitan locations.

## 5. Limitations

This review has several limitations. Only studies published in English and the peer-reviewed literature were included, which may have excluded relevant evidence from non-English publications and the grey literature. The exclusion of refugees, asylum seekers, children and adolescents limits the applicability of the findings to these migrant groups. Most included studies were conducted in metropolitan settings, which may not reflect the experiences of migrants living in regional, rural, or remote areas of Australia. As a scoping review, the quality of included studies was not critically appraised.

## 6. Conclusions

This scoping review highlights the diverse primary healthcare needs of migrants in Australia and demonstrates that equitable access extends beyond service availability. Effective primary healthcare requires culturally responsive communication, improved health literacy, accessible interpreter services, continuity of care and models of care that recognise the diversity of migrant communities. Strengthening these aspects of primary healthcare has the potential to improve preventative care, enhance patient engagement and reduce inequities in health outcomes. Future research should evaluate interventions that improve culturally responsive primary healthcare and identify scalable approaches that can be implemented across diverse Australian healthcare settings.

## Figures and Tables

**Figure 1:**
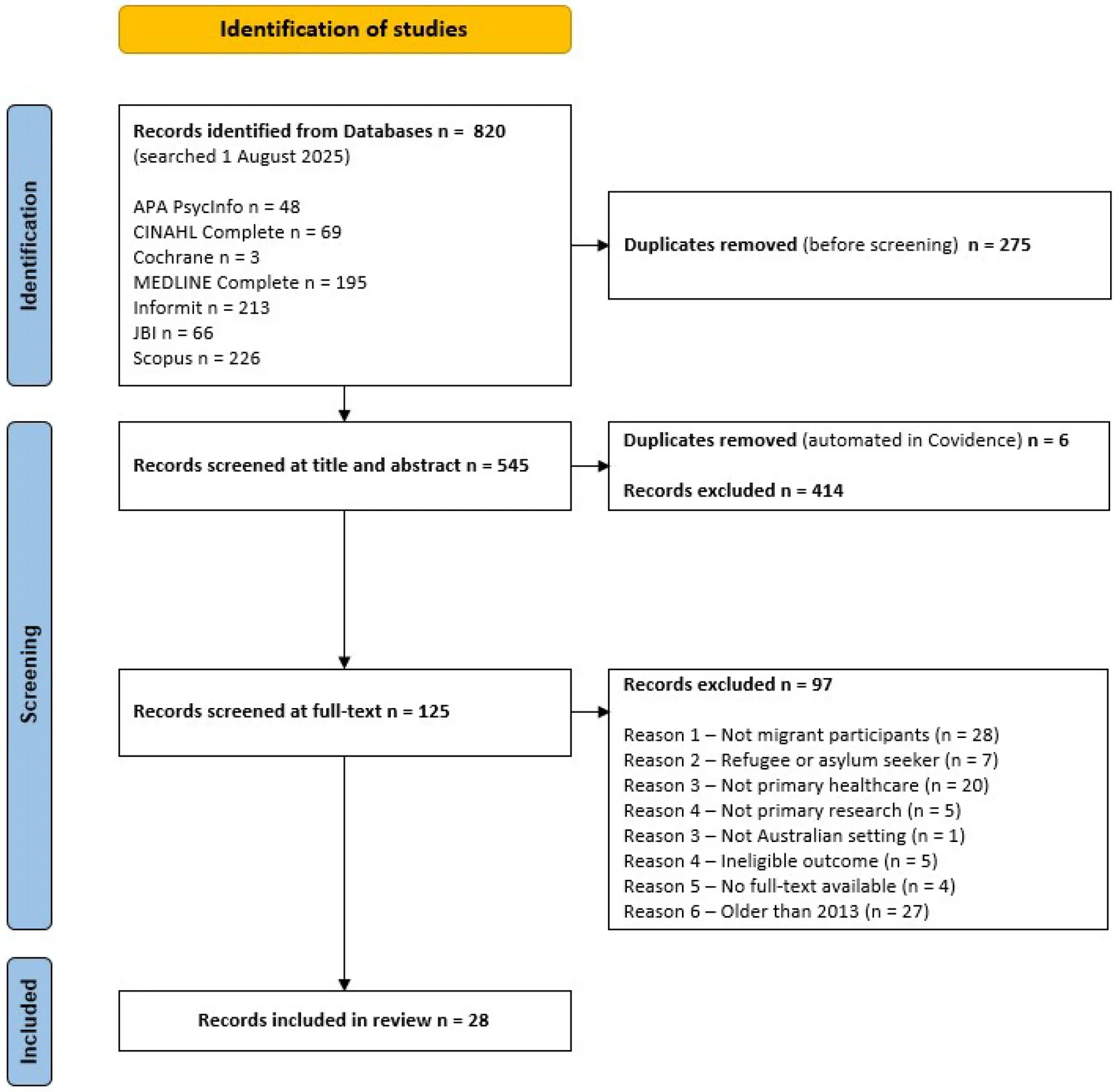
PRISMA flow diagram.

**Table 1:** Study inclusion and exclusion criteria using the Population, Concept, Context framework.

Component	Inclusion	Exclusion
**Population**	Adult migrants (aged ≥18 years) regardless of gender, who access primary healthcare services in Australia.	Refugees, asylum seekers, studies focused on children or adolescents (<18 yrs). Studies where migrant-specific findings could not be distinguished. *
**Concept**	Studies examining migrants’ needs, access or utilisation of any component of primary healthcare.	Studies examining health outcomes only, without discussion of primary healthcare needs, access, utilisation, or experiences.
**Context**	Primary healthcare services within the Australian system, including general practice, community, preventative healthcare, health promotion and other community-based primary healthcare services.	Studies conducted outside Australia or those not focused on primary healthcare settings, for example, studies focused solely on secondary, tertiary, hospital-based, or specialist healthcare.
**Study Design**	Primary research using qualitative, quantitative or mixed-methods designs.	Literature reviews, systematic reviews, scoping reviews, protocols, editorials, commentaries, opinion pieces, conference abstracts, dissertations and grey literature.
**Publication Characteristics**	Peer-reviewed articles in English between 2013 and 2025.	Non-peer-reviewed publications in English and studies published prior to 2013.
**Outcome Focus**	Studies reporting migrants’ reasons for and experiences with accessing primary healthcare services.	Studies not reporting findings relevant to engagement with and experiences of primary healthcare services.

Note: * Refugees, asylum seekers, children, and adolescents were excluded because these populations may have distinct healthcare needs, service access pathways, and healthcare experiences that differ from those of adult migrants. To maintain a clearly defined review scope, this review focused on adult migrants.

## Data Availability

No new data were created or analyzed in this study. Data sharing is not applicable to this article.

## Supplementary Materials

The following supporting information can be downloaded at: https://www.mdpi.com/article/10.3390/healthcare14193168/s1, Table S1: Preferred Reporting Items for Systematic reviews and Meta-Analyses extension for Scoping Reviews (PRISMA-ScR) checklist; Table S2: Study characteristics.

## References

[1] Australian Bureau of Statistics (ABS) (2026). Australia’s Population by Country of Birth. https://www.abs.gov.au/statistics/people/population/australias-population-country-birth/latest-release.

[2] Federal Court of Australia (2025). Migration Law Guide: Introduction to Migration. https://www.fedcourt.gov.au/law-and-practice/guides/migration/introduction#:~:text=Migration%20is%20the%20movement%20of,intention%20of%20remaining%20there%20permanently.

[3] Acquadro-Pacera G., Valente M., Facci G., Kiros B.M., Della Corte F., Barone-Adesi F., Ragazzoni L., Trentin M. (2024). Exploring differences in the utilization of the emergency department between migrant and non-migrant populations: A systematic review. BMC Public Health.

[4] McBride J., Block A., Russo A. (2017). An integrated healthcare service for asylum seekers and refugees in the South-Eastern Region of Melbourne: Monash Health Refugee Health and Wellbeing. Aust. J. Prim. Health.

[5] Khatri R.B., Assefa Y. (2022). Access to health services among culturally and linguistically diverse populations in the Australian universal health care system: Issues and challenges. BMC Public Health.

[6] Patel P., Bernays S., Dolan H., Muscat D.M., Trevena L. (2021). Communication Experiences in Primary Healthcare with Refugees and Asylum Seekers: A Literature Review and Narrative Synthesis. Int. J. Environ. Res. Public Health.

[7] World Health Organization (WHO) (2023). Primary Health Care. https://dev-cms.who.int/news-room/fact-sheets/detail/primary-health-care.

[8] National Rural Health Alliance (NRHA) Latest Snapshot of Rural Health Out Now! 2025. https://www.ruralhealth.org.au/nrhanews/latest-snapshot-of-rural-health-out-now/.

[9] Moezzi S.M.I., Etemadi M., Lankarani K.B., Behzadifar M., Katebzada H., Shahabi S. (2024). Barriers and facilitators to primary healthcare utilization among immigrants and refugees of low and middle-income countries: A scoping review. Glob. Health.

[10] Tsai P.L., Ghahari S. (2023). Immigrants’ Experience of Health Care Access in Canada: A Recent Scoping Review. J. Immigr. Minor Health.

[11] Adhikari M., Kaphle S., Dhakal Y., Duwadi S., Subedi R., Shakya S., Tamang S., Khadka M. (2021). Too long to wait: South Asian migrants’ experiences of accessing health care in Australia. BMC Public Health.

[12] Bandyopadhyay M. (2021). Gestational diabetes mellitus: A qualitative study of lived experiences of South Asian immigrant women and perspectives of their health care providers in Melbourne, Australia. BMC Pregnancy Childbirth.

[13] Javanparast S., Naqvi S.K.A., Mwanri L. (2020). Health service access and utilisation amongst culturally and linguistically diverse populations in regional South Australia: A qualitative study. Rural Remote Health.

[14] Peprah P., Lloyd J., Harris M. (2023). Health literacy and cultural responsiveness of primary health care systems and services in Australia: Reflections from service providers, stakeholders, and people from refugee backgrounds. BMC Public Health.

[15] Saito S., Harris M.F., Long K.M., Lewis V., Casey S., Hogg W., Cheng I.-H., Advocat J., Marsh G., Gunatillaka N. (2021). Response to language barriers with patients from refugee background in general practice in Australia: Findings from the OPTIMISE study. BMC Health Serv. Res..

[16] White J., Plompen T., Tao L., Micallef E., Haines T. (2019). What is needed in culturally competent healthcare systems? A qualitative exploration of culturally diverse patients and professional interpreters in an Australian healthcare setting. BMC Public Health.

[17] Kamran H., Hassan H., Ali M.U.N., Ali D., Taj M., Mir Z., Pandya M., Steinberg S.R., Jamal A., Zaidi M. (2022). Scoping review: Barriers to primary care access experienced by immigrants and refugees in English-speaking countries. Qual. Res. J..

[18] Lakin K., Kane S. (2023). A critical interpretive synthesis of migrants’ experiences of the Australian health system. Int. J. Equity Health.

[19] Arksey H., O’Malley L. (2005). Scoping studies: Towards a methodological framework. Int. J. Soc. Res. Methodol..

[20] Levac D., Colquhoun H., O’Brien K.K. (2010). Scoping studies: Advancing the methodology. Implement Sci..

[21] Tricco A.C., Lillie E., Zarin W., O’Brien K.K., Colquhoun H., Levac D., Moher D., Peters M.D.J., Horsley T., Weeks L. (2018). PRISMA Extension for Scoping Reviews (PRISMA-ScR): Checklist and Explanation. Ann. Intern Med..

[22] Aromataris E., Lockwood C., Porritt K., Pilla B., Jordan Z. (2024). JBI Manual for Evidence Synthesis. JBI. https://synthesismanual.jbi.global.

[23] Munn Z., Peters M.D.J., Stern C., Tufanaru C., McArthur A., Aromataris E. (2018). Systematic review or scoping review? Guidance for authors when choosing between a systematic or scoping review approach. BMC Med. Res. Methodol..

[24] Commonwealth of Australia (2013). National Primary Health Care Strategic Framework. Commonwealth of Australia. https://www.parliament.qld.gov.au/Work-of-the-Assembly/Tabled-Papers/docs/5413t3298/5413t3298.pdf.

[25] Naeem M., Ozuem W., Howell K., Ranfagni S. (2023). A Step-by-Step Process of Thematic Analysis to Develop a Conceptual Model in Qualitative Research. Int. J. Qual. Methods.

[26] Nowell L.S., Norris J.M., White D.E., Moules N.J. (2017). Thematic analysis: Striving to meet the trustworthiness criteria. Int. J. Qual. Methods.

[27] Ahmad A., Khan M.U., Aslani P. (2021). A Qualitative Study on Medication Taking Behaviour Among People with Diabetes in Australia. Front Pharmacol..

[28] Alzayer R., Chaar B., Basheti I., Saini B. (2018). Asthma management experiences of Australians who are native Arabic speakers. J. Asthma.

[29] Alzubaidi H., Mc Mamara K., Chapman C., Stevenson V., Marriott J. (2015). Medicine-taking experiences and associated factors: Comparison between Arabic-speaking and Caucasian English-speaking patients with Type 2 diabetes. Diabet. Med..

[30] Alzubaidi H., Mc Namara K., Browning C. (2017). Time to question diabetes self-management support for Arabic-speaking migrants: Exploring a new model of care. Diabet. Med..

[31] Alzubaidi H., Mc Namara K., Browning C., Marriott J. (2015). Barriers and enablers to healthcare access and use among Arabic-speaking and Caucasian English-speaking patients with type 2 diabetes mellitus: A qualitative comparative study. BMJ Open.

[32] Blignault I., Saab H., Youssef H., Baddah H., Giourgas K., Woodland L. (2023). “It Is Definitely a Good Program for Everyone from Every Community”: A Qualitative Study of Community Partner Perspectives on the Culturally and Linguistically Diverse (CALD) Mindfulness Program. Int. J. Environ. Res. Public Health.

[33] Gray C., Lobo R., Narciso L., Oudih E., Gunaratnam P., Thorpe R., Crawford G. (2019). Why I Can’t, Won’t or Don’t Test for HIV: Insights from Australian Migrants Born in Sub-Saharan Africa, Southeast Asia and Northeast Asia. Int. J. Environ. Res. Public Health.

[34] Hurley C., Panagiotopoulos G., Tsianikas M., Newman L., Walker R. (2013). Access and acceptability of community-based services for older Greek migrants in Australia: User and provider perspectives. Health Soc. Care Community.

[35] Inoue K., Kelly M., Bateson D., Rutherford A., Stewart M., Richters J. (2016). Contraceptive choices and sexual health of Japanese women living in Australia: A brief report from a qualitative study. Aust. Fam. Physician.

[36] Istiko S.N., Remata S., Ndayizeye A., Moreno M.E.V., Kirunda V., Hollingdrake O., Osborne R., Hou J.Z., Abell B., Mullens A.B. (2024). Developing critical HIV health literacy: Insights from interviews with priority migrant communities in Queensland, Australia. Cult. Health Sex..

[37] Maheen H., Damabi N.M., Lassi Z.S. (2025). Improving engagement with sexual and reproductive health services among young African migrants in Australia. Sex. Health.

[38] Raymundo G., Smith-Merry J., McNab J. (2021). Experiences of health service literacy and access amongst Australian young adults from migrant backgrounds. Health Promot. J. Aust..

[39] Robotin M.C., Wallace J., Gallego G., George J. (2021). Hepatitis B and Liver Cancer: Community Awareness, Knowledge and Beliefs of Middle Eastern Migrants in Sydney, Australia. Int. J. Environ. Res. Public Health.

[40] Stanzel K.A., Hammarberg K., Nguyen T., Fisher J. (2022). ‘They should come forward with the information’: Menopause-related health literacy and health care experiences among Vietnamese-born women in Melbourne, Australia. Ethn. Health.

[41] Vatcharavongvan P., Hepworth J., Lim J., Marley J. (2014). What are the health needs, familial and social problems of Thai migrants in a local community in Australia? A focus group study. J. Immigr. Minor Health.

[42] Xiao L.D., Tu Q., Bickford J., Muir-Cochrane E., Martino T. (2023). The Experiences of Older Chinese Migrants with Chronic Diseases during COVID-19 Pandemic in Australia. Health Soc. Care Community.

[43] Zeng L., Xu X., Perry L., Skouteris H. (2024). The Role of Acculturation in Self-Care Behaviours among Chinese Immigrants Living with Cardiovascular Disease: A Qualitative Study. Health Soc. Care Community.

[44] Alam Z., Ann Dean J., Janda M. (2022). Cervical screening uptake: A cross-sectional study of self-reported screening attitudes, behaviours and barriers to participation among South Asian immigrant women living in Australia. Womens Health.

[45] Alam Z., Dean J.A., Janda M. (2022). What do South Asian immigrant women know about HPV, cervical cancer and its early detection: A cross-sectional Australian study. J. Migr. Health.

[46] Brown H., Breislin E. (2024). Comparing health service usage of migrant groups in Australia: Evidence from the household income and labour dynamics survey of Australia. J. Migr. Health.

[47] Hu J., Wang Z. (2016). Exploring the associated factors of elevated psychological distress in a community residing sample of Australian Chinese migrants. Aust. J. Psychol..

[48] Hu J., Wang Z. (2016). Non-prescribed antibiotic use and general practitioner service utilisation among Chinese migrants in Australia. Aust. J. Prim. Health.

[49] Ruwanpathirana T., Reid C.M., Owen A.J., Fong D.P., Gowda U., Renzaho A.M. (2014). Assessment of vitamin D and its association with cardiovascular disease risk factors in an adult migrant population: An audit of patient records at a Community Health Centre in Kensington, Melbourne, Australia. BMC Cardiovasc. Disord..

[50] Sanchez L., Johnson T., Williams S., Spurling G., Durham J. (2020). Identifying inequities in an urban Latin American population: A cross-sectional study in Australian primary health care. Aust. J. Prim. Health.

[51] Saraswat N., Everett B., Pillay R., Prabhu N., Villarosa A., George A. (2022). Knowledge, Attitudes and Practices of Indian Immigrants in Australia towards Oral Cancer and Their Perceived Role of General Practitioners: A Cross-Sectional Study. Int. J. Environ. Res. Public Health.

[52] Taye B.W., Valery P.C., Liddle B., Woodward A.J., Sackey D., Williams S., Chang G.K.F., Clark P.J. (2022). Fitting Health Care to People: Understanding and Adapting to the Epidemiology and Health Literacy of People Affected by Viral Hepatitis from Culturally and Linguistically Diverse Migrant Backgrounds. J. Immigr. Minor Health.

[53] Ramanathan V., Sitharthan G., Pepper K., Wylie K. (2013). Sexual health of Indian immigrant men in Australia: An exploratory research on help-seeking attitudes. Sex. Health.

[54] Stanzel K., Pham M., Hammarberg K., Fisher J. (2025). Midlife care to migrant women: Primary healthcare providers’ beliefs about barriers and facilitators. Prim. Health Care Res. Dev..

[55] Sassen B. (2023). Improving Person-Centered Innovation of Nursing Care: Leadership for Change.

[56] Fauk N.K., Ziersch A., Gesesew H., Ward P., Green E., Oudih E., Tahir R., Mwanri L. (2021). Migrants and Service Providers’ Perspectives of Barriers to Accessing Mental Health Services in South Australia: A Case of African Migrants with a Refugee Background in South Australia. Int. J. Environ. Res. Public Health.

[57] Joshi C., Russell G., Cheng I.-H., Kay M., Pottie K., Alston M., Smith M., Chan B., Vasi S., Lo W. (2013). A narrative synthesis of the impact of primary health care delivery models for refugees in resettlement countries on access, quality and coordination. Int. J. Equity Health.

[58] Schmidt N.C., Fargnoli V., Epiney M., Irion O. (2018). Barriers to reproductive health care for migrant women in Geneva: A qualitative study. Reprod. Health.

[59] Schouten B.C., Cox A., Duran G., Kerremans K., Banning L.K., Lahdidioui A., Muijsenbergh M.v.D., Schinkel S., Sungur H., Suurmond J. (2020). Mitigating language and cultural barriers in healthcare communication: Toward a holistic approach. Patient Educ. Couns..

[60] Singla R., Perumbil Pathrose S., Lane A.P., Ogunsiji O. (2026). Primary health care experiences of Indian immigrants living with chronic illnesses in Australia: A qualitative study. Prim. Health Care Res. Dev..

[61] Topçu M.-T., Mösko M. (2025). Building trust and relationship across language barriers: A qualitative study of interpreter-mediated psychotherapy with therapists, interpreters and patients. Int. J. Equity Health.

[62] Brooks L.A., Manias E., Bloomer M.J. (2019). Culturally sensitive communication in healthcare: A concept analysis. Collegian.

[63] Williams N., Griffin G., Wall M., Bradfield Z. (2025). The sexual and reproductive health knowledge of women in Australia: A scoping review. Health Promot. J. Austr..

[64] Bin Abdul Baten R., Thapa K., Saadi A. (2026). Access to care and self-rated health status: Comparison of rural and urban immigrants in the US. J. Immigr. Minor Health.

[65] Zeng Y., Kang X., Yang Y., Hwang E. (2025). Barriers and facilitators of preventive healthcare access among immigrants in rural America: A scoping review. Int. J. Equity Health.

