# Supplementary material for: Exploring Primary Healthcare Needs Among Migrants in Australia: A Scoping Review
Source: Healthcare (Basel). 2026 Sep 24;14(19):3168. doi: 10.3390/healthcare14193168 (PMC13664834; doi:10.3390/healthcare14193168)
Supplement: Supplementary file 1 [file healthcare-14-03168-s001.zip › Table S2.pdf]

Exploring primary healthcare needs among Australian migrants: A scoping review

Table S2 – Study Characteristics

First Author and Year	Title	Location	Population	Design	Healthcare focus	Key findings related to migrants' primary healthcare needs and experiences
Ahmad 2021	A Qualitative Study on Medication Taking Behaviour Among People With Diabetes in Australia	New South Wales	Indian migrants with diagnosed with Type 2 diabetes and using antidiabetic medicine (Indian born, living in Sydney, speaks fluent English)	Qualitative study	Diabetes management, medication adherence, education about diabetes	• Medication adherence varied across the three phases<br>• Barriers included fear of side effects, cultural beliefs, and lack of understanding<br>• Enablers included GP support, desire to improve Health outcomes, and access to healthcare services
Alam 2022	Cervical screening uptake: A cross-sectional study of self-reported screening attitudes, behaviours and barriers to participation among South Asian immigrant women living in Australia	Queensland	South Asian immigrant women living in Queensland	Cross-sectional quantitative study	Cervical cancer screening (Pap test/HPV test)	• Increased screening uptake and improved knowledge with longer residency<br>• Identification of key behavioural and informational barriers
Alam 2022	What do South Asian immigrant women know about HPV, cervical	Queensland	South Asian immigrant	Cross-sectional observational	Cervical cancer screening, HPV awareness,	• Identification of knowledge gaps

First Author and Year	Title	Location	Population	Design	Healthcare focus	Key findings related to migrants' primary healthcare needs and experiences
	cancer and its early detection: A cross-sectional Australian study		women living in Queensland	quantitative study	education on screening methods	• Association between knowledge and screening participation<br>• Need for culturally tailored education
Alzayer 2018	Asthma management experiences of Australians who are native Arabic speakers	Melbourne	Arabic speaking people with asthma or carers of those with asthma with low English proficiency around Melbourne Australia	Qualitative study	Asthma management, education, awareness and culturally appropriate care	• Five key themes: (1) stigma; (2) health literacy; (3) treatment non-adherence; (4) unrealistic expectations; and (5) coping style issues<br>• Participants reported low asthma control, poor Health literacy, cultural beliefs affecting treatment adherence, and limited engagement with healthcare services
Alzubaidi 2015	Medicine-taking experiences and associated factors: comparison between Arabic-speaking and Caucasian English-	Victoria	Adults with Type 2 diabetes	Qualitative study	Diabetes management, medication adherence, culturally	• Arabic-speaking participants often modified or rejected prescribed regimens, influenced by cultural beliefs, stigma, and peer advice

First Author and Year	Title	Location	Population	Design	Healthcare focus	Key findings related to migrants' primary healthcare needs and experiences
	speaking patients with Type 2 diabetes				appropriate education	• Arabic-speaking Participants reported poor communication with healthcare providers and minimal engagement with pharmacists
Alzubaidi 2015	Barriers and enablers to healthcare access and use among Arabic-speaking and Caucasian English-speaking patients with type 2 diabetes mellitus: a qualitative comparative study	Victoria	Caucasian English-speaking and Arabic-speaking healthcare recipients with diabetes	Qualitative study	Diabetes diagnosis, management, education, culturally appropriate care	• Arabic-speaking migrants intentionally delayed seeking healthcare even when symptomatic<br>• Four key barriers and enablers identified: (1) influence of significant others; (2) sociocultural and religious beliefs; (3) experiences with healthcare providers; and (4) lack of knowledge about healthcare services<br>• Delayed diagnosis and treatment due to cultural and religious beliefs (Arabic-speaking participants influenced by significant others, religious beliefs,

First Author and Year	Title	Location	Population	Design	Healthcare focus	Key findings related to migrants' primary healthcare needs and experiences
						previous services experiences)
Alzubaidi 2017	Time to question diabetes self-management support for Arabic-speaking migrants: exploring a new model of care	Victoria	First-generation Arabic-speaking migrants with Type 2 diabetes	Qualitative study	Diabetes self-management education, emotional support, culturally appropriate care	• No ongoing self-management support was offered beyond initial diagnosis<br>• Participants had significant knowledge gaps and emotional distress<br>• Strong preference for face-to-face, storytelling-based education in gender-specific groups<br>• Technology-based interventions were poorly accepted<br>• Desire for culturally relevant education and support
Blignault 2023	"It Is Definitely a Good Program for Everyone from Every Community": A Qualitative Study of Community Partner Perspectives on the	Sydney	Community partners (clinicians and community workers) involved in	Qualitative study	Mental health support, stigma reduction, culturally appropriate care	• The program was perceived as positive and rewarding, culturally inclusive, impactful, and empowering<br>• Improved mental health and well-being, reduced stigma,

First Author and Year	Title	Location	Population	Design	Healthcare focus	Key findings related to migrants' primary healthcare needs and experiences
	Culturally and Linguistically Diverse (CALD) Mindfulness Program		delivering mindfulness programs to Arabic and Bangla-speaking communities			and increased community engagement and trust<br>• Trust, safety, and cultural tailoring were critical to success
Brown 2024	Comparing health service usage of migrant groups in Australia: Evidence from the household income and labour dynamics survey of Australia	National	Migrants in Australia (aged 16 years and over)	Quantitative study	Routine care and preventative screenings	• Migrant groups show varied patterns of service usage (younger women from South Asia, Latin America, and Eastern/Southern Europe, and younger men from Eastern/Southern Europe had lower GP visit rates)<br>• Higher income and qualifications resulted in lower interactions with healthcare teams<br>• Older immigrants more likely to use services than younger
Gray 2019	Why I Can't, Won't or Don't Test for HIV: Insights from Australian	Adelaide	Migrants from sub-Saharan African,	Qualitative study	Confidential, accessible, culturally	• Low uptake of HIV testing due to stigma and system navigation issues (cost,

First Author and Year	Title	Location	Population	Design	Healthcare focus	Key findings related to migrants' primary healthcare needs and experiences
	Migrants Born in Sub-Saharan Africa, Southeast Asia and Northeast Asia		Southeast Asia and Northeast Asia living in Australia		appropriate HIV testing	eligibility, stigma, low visibility of HIV, and missed opportunities by GPs)
Hu 2016	Exploring the associated factors of elevated psychological distress in a community residing sample of Australian Chinese migrants	National	Chinese migrants residing in Australia	Cross-sectional, exploratory quantitative study	Mental health support for psychological distress	• Associations between psychological distress and healthcare satisfaction<br>• Risk factors for psychological distress included younger age, being single/divorced, student status, lower education, shorter duration in Australia (<5 years), lower English proficiency
Hu 2016	Non-prescribed antibiotic use and general practitioner service utilisation among Chinese migrants in Australia	National	Chinese migrants living in Australia	Cross-sectional quantitative study	Access to antibiotics and primary care	• Participants satisfied with GP services were significantly less likely to self-medicate
Hurley 2013	Access and acceptability of community-based services for older Greek migrants in Australia:	Adelaide	Greek Elders, their carers and staff from Greek	Qualitative descriptive study	Support to age in place, maintain independence, and	• Older Greek migrants face barriers to accessing formal services (language, cost,

First Author and Year	Title	Location	Population	Design	Healthcare focus	Key findings related to migrants' primary healthcare needs and experiences
	user and provider perspectives		organisations/ service providers		reduce institutionalisation	transport, and cultural attitudes)<br>• Strong reliance on family for both informal care and as mediators with formal services<br>• Many elders are unaware of available services (unless already linked through family or health professionals)<br>• Service providers often lack bilingual staff and struggle to promote services effectively to this group<br>• Cultural expectations of family care persist, sometimes delaying formal service uptake until emergencies arise
Inoue 2016	Contraceptive choices and sexual health of Japanese women living in	New South Wales	Japanese migrant women	Qualitative study	Contraceptive access, sexual health support, sexually	• Continued reliance on culturally familiar contraceptive methods<br>• Limited engagement with formal sexual health services

First Author and Year	Title	Location	Population	Design	Healthcare focus	Key findings related to migrants' primary healthcare needs and experiences
	Australia: A brief report from a qualitative study				transmitted infection screening	• Limited use of the combined oral contraceptive pill, often due to cultural attitudes and language barriers
Istiko 2024	Developing critical HIV health literacy: insights from interviews with priority migrant communities in Queensland, Australia	Queensland	Priority migrant communities in Southeast Queensland	Qualitative study	HIV prevention, testing, treatment, and sexual health education	• Improved engagement with HIV Health services<br>• HIV Health literacy is relational, contextual, and influenced by socio-cultural norms<br>• Participants used relational strategies (e.g., partnerships with GPs, literacy mediators) to appraise and apply HIV health information<br>• Barriers to HIV Health literacy include stigma, lack of culturally competent services, and systemic gaps in education and healthcare integration<br>• Participants demonstrated agency and reflective capacity

First Author and Year	Title	Location	Population	Design	Healthcare focus	Key findings related to migrants' primary healthcare needs and experiences
						in navigating health systems and information
Maheen 2025	Improving engagement with sexual and reproductive health services among young African migrants in Australia	Melbourne	Young African migrants in Melbourne	Qualitative study	Confidential, culturally sensitive SRH care	• Three key themes: (1) lack of visibility and information of existing sexual health services affects service use; (2) cultural stigma associated with sexual health affects help-seeking and health service engagement (increased vulnerability to poor sexual health outcomes); and (3) key attributes of youth-friendly sexual and reproductive healthcare for young African migrants
Ramanathan 2013	Sexual health of Indian immigrant men in Australia: an exploratory research on help-seeking attitudes	National	Adult Indian immigrants	Mixed-methods sequential exploratory design	Sexual health	• Reported help-seeking intentions/preferences (high stated willingness to seek medical help)<br>• Age-related differences in specialist vs GP preference

First Author and Year	Title	Location	Population	Design	Healthcare focus	Key findings related to migrants' primary healthcare needs and experiences
						(Doctor gender/ethnicity largely not important)
Raymundo 2021	Experiences of health service literacy and access amongst Australian young adults from migrant backgrounds	New South Wales	25 young people with migrant backgrounds living in Greater Western Sydney	Qualitative study	Preventative care, to address symptoms of illnesses, or in conjunction with western medicine	• Barriers: Poor youth-friendliness of services, stigma around mental/sexual health, low health literacy, long wait times, lack of flexible hours<br>• Facilitators: Positive rapport, confidentiality, cultural/linguistic concordance, family/community support
Robotin 2021	Hepatitis B and Liver Cancer: Community Awareness, Knowledge and Beliefs of Middle Eastern Migrants in Sydney, Australia	Sydney	Arabic- and Assyrian-speaking migrant communities, community leaders and members	Qualitative study	Viral hepatitis and liver cancer knowledge, other broad healthcare needs	• Limited knowledge of conditions and stigma associated with both<br>• Variable health literacy levels and trust in medical professionals<br>• Fear that receiving "bad news" would deter people from seeking care

First Author and Year	Title	Location	Population	Design	Healthcare focus	Key findings related to migrants' primary healthcare needs and experiences
						• Preferred family doctors, family members, the internet and the ethnic media for health information
Ruwanpathirana 2014	Assessment of vitamin D and its association with cardiovascular disease risk factors in an adult migrant population: an audit of patient records at a Community Health Centre in Kensington, Melbourne, Australia	Melbourne	Adults attending Community Health Centre from 1 January 2010 to 31 December 2012	Cross-sectional clinical audit of electronic medical records	Vitamin D testing/management and cardiovascular risk assessment in primary care	• Centre prioritized Vitamin D deficiency testing for migrants, middle-aged, females, and those with diabetes/osteoarthritis<br>• Higher risk of Vitamin D deficiency among migrants from low-latitude origins
Sanchez 2020	Identifying inequities in an urban Latin American population: a cross-sectional study in Australian primary health care	Brisbane	382 Spanish-speaking Latin American migrants	Cross-sectional quantitative study	Cardiometabolic risk reduction (dyslipidaemia, hypertension, diabetes, obesity/ overweight); musculoskeletal care; mental health support; comprehensive chronic disease	• Identification of gaps (e.g. under-utilisation of management plans), risk factors, service use and morbidity to inform targeted improvements in culturally responsive primary care

First Author and Year	Title	Location	Population	Design	Healthcare focus	Key findings related to migrants' primary healthcare needs and experiences
					management, health assessments, and yearly diabetes care visit.	
Saraswat 2022	Knowledge, Attitudes and Practices of Indian Immigrants in Australia towards Oral Cancer and Their Perceived Role of General Practitioners: A Cross-Sectional Study	National	164 Indian immigrants	Cross-sectional quantitative study	Oral health knowledge and practices	• Gaps in knowledge of alcohol and areca/betel risks, key symptoms, and GP screening<br>• Most had seen a GP/clinic in the past year (73%) but far fewer had seen a dentist (35%) (only 12% recalled receiving oral cancer information during healthcare visits)<br>• Most believed GPs can help detect oral problems, were open to GP oral-health advice, and would attend a dentist if referred by a GP<br>• Preventive behaviours were widely valued, and many preferred culturally

First Author and Year	Title	Location	Population	Design	Healthcare focus	Key findings related to migrants' primary healthcare needs and experiences
						concordant clinicians for routine check-ups
Stanzel 2022	'They should come forward with the information': menopause-related health literacy and health care experiences among Vietnamese-born women in Melbourne, Australia	Melbourne	Vietnamese-born immigrant women in Melbourne	Qualitative study	Accessible menopause information (Vietnamese language), GP-initiated counselling on symptoms, risks, and healthy behaviours, support navigating screening results	• Limited English proficiency constrained health-literacy competencies (access, understanding, evaluation, use)<br>• More information sourced from family/ friends (limited independent information seeking)
Stanzel 2025	Midlife care to migrant women: primary healthcare providers' beliefs about barriers and facilitators	National	76 primary healthcare providers in Australia delivering midlife care to migrant women	Cross-sectional mixed-methods study	Culturally competent midlife health promotion and preventive care for migrant women (screening; counselling on exercise/diet; symptom	• Migration/settlement priorities (family, finances, language, co-morbidities) often de-prioritise menopause discussions

First Author and Year	Title	Location	Population	Design	Healthcare focus	Key findings related to migrants' primary healthcare needs and experiences
					management; navigation)	
Taye 2022	Fitting Health Care to People: Understanding and Adapting to the Epidemiology and Health Literacy of People Affected by Viral Hepatitis from Culturally and Linguistically Diverse Migrant Backgrounds	Brisbane	66 Culturally and Linguistically Diverse migrant adults with Hepatitis B Virus and Hepatitis C Virus in Brisbane	Prospective cohort study (quantitative)	Improve viral hepatitis literacy, dispel misconceptions, promote screening, treatment uptake, and liver cancer surveillance, and address cultural and linguistic barriers	• Identified literacy gaps and structural barriers<br>• Recommended culturally adapted care models and health information delivery strategies
Vatcharavongvan 2014	What are the health needs, familial and social problems of Thai migrants in a local community in Australia? A focus group study	Brisbane	20 Thai migrants in Brisbane	Qualitative study	General physical health concerns	• Identified complex, culture-bound problems, highlighted Temple/ cultural systems as both risks and resources, underscoring need for culturally responsive service design<br>• Use of complementary and alternative medicines

First Author and Year	Title	Location	Population	Design	Healthcare focus	Key findings related to migrants' primary healthcare needs and experiences
						• Health information sought from internet, books, Thai friends
Xiao 2023	The Experiences of Older Chinese Migrants with Chronic Diseases during COVID-19 Pandemic in Australia	National	Chinese migrants with non-communicable chronic diseases and family carers	Qualitative descriptive study	Management of chronic diseases (hypertension, diabetes, coronary heart disease, arthritis), access to GP services, interpreter support, and culturally appropriate care	• Loss of social capital and neighbourhood cohesion and worsened mental health<br>• Increased health inequalities due to language barriers and telehealth challenges<br>• Bilingual/ usual GP services or telehealth available (or carer available for translation) improved experiences
Zeng 2024	The Role of Acculturation in Self-Care Behaviours among Chinese Immigrants Living with Cardiovascular Disease: A Qualitative Study	Sydney	20 First-generation Chinese immigrants with cardiovascular disease living in Australia	Qualitative descriptive study	Self-care behaviours for cardiovascular disease	• Acculturation influenced self-care in four domains: cultural practices (dietary habits, social networks, language/ media), emotional well-being influenced by family dynamics and cultural values (traditional beliefs, family ties), healthcare system

First Author and Year	Title	Location	Population	Design	Healthcare focus	Key findings related to migrants' primary healthcare needs and experiences
						navigation, and new living environment<br>• Language barriers limited access to acute care and heart health information<br>• Traditional Chinese Medicine (TCM) beliefs complicated medication adherence and acute symptom management<br>• Community associations enabled exercise and health education

GP: General Practitioner; HIV: Human Immunodeficiency Virus
